# 

**Published:** 1892-02-20

**Authors:** 


PRICE ONE PENNY.

282, Vol. XI.—February 20th, 1892.

Registered at the General Post Office as a Newspaper for transmission in the United Kingdom and Abroad.]

### THE HOSPITAL

WITH EXTRA NURSING SUPPLEMENT
Per Annum, 6s. 6d., post free.
Monthly Parts, 6d.; post free, 8d.
Ditto per Annum, 8s., post free

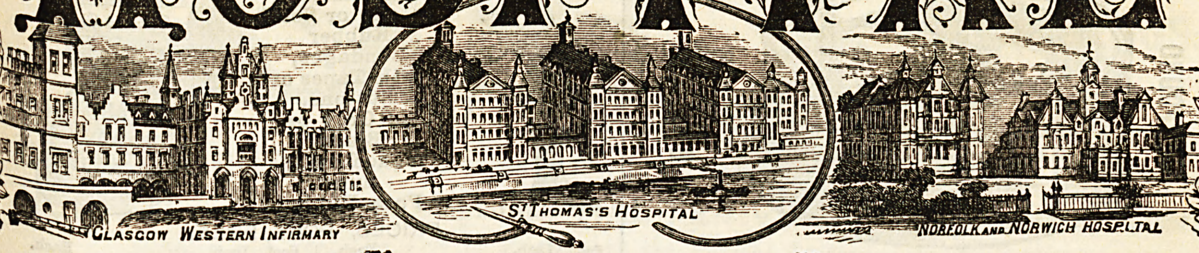

A WEEKLY JOURNAL OF

### Science, Medicine, Nursing, and Philanthropy.

SATURDAY, FEBRUARY 20th, 1892.

**Salvationism and the Care of the Sick** - 249
**Passing Topics.**—Fogs - 250
**The Doctor's Armchair.**—Ungrateful Patients - 251
**Some Scientific Aspects of Insanity.** XIII.—Dementia 252
**Some American Hospitals.** VIII.—The University of Pennsylvania Hospital. IX.—Garfield Memorial Hospital, Washington - 253
**Everybody's Column** - 253
**Notes and News** - 254
**Annotations.**—The Lords and the London Fogs—The Training of Child Acrobats—Universities and their Labels - 256
**General Charities—Scraps and Gleanings** - 255
**The Practitioner's Mirror and Retrospect—**
Medicine.—Gastric Hyperæsthesia and Acidity - 257
Surgery.—Tubercular Ulcers of the Tongue - 258
New Drugs, Appliances, and Things Medical.—Spiking's Malt Nursery Biscuits — Celery Coffee — Liquor Carnis Suppositories — Newly Invented Spinal Chair — Ammoniated Tincture of Quinine - 259
**The Ticket System** - 259
**A Physiologist's View of Smoking** - 260
**The Student of Medicine.**—Appointments—Vacancies

EXTRA SUPPLEMENT.—THE NURSING MIRROR.

**En Passant.**—Another Home—Short Items—Workhouse Infirmary Nurses—Women as Army Nurses—American District Nurses—Canterbury Institute - cxxi

**Lectures on Surgical Ward Work and Nursing.** By Alex. Miles, M.D.Edin., F.R.C.S.E. Lecture XLVI.—Instruments - cxxii

**Appointments** - cxxiii

**Presentation** - cxxiii

**Wants and Workers** - cxxiii

**For Reading to the Sick.**—Rose-coloured Curtains - cxxiii

**District Nurses under the Poor Law** - cxxiv

**Everybody's Opinion.**—North London Nursing Association—A Nursing Reserve—One Funeral Makes Many - cxxiv

**Memoranda for Matrons** - cxxv

**The Mortality of German Catholic Nurses** - cxxv

**Off Duty.**—Saint and Sinner - cxxvi

**Where to Go** - cxxvi

**Notes and Queries** - cxxvi

